# Balance training can enhance hip fracture patients’ independence in activities of daily living: A meta-analysis of randomized controlled trials

**DOI:** 10.1097/MD.0000000000019641

**Published:** 2020-04-17

**Authors:** Xinxin Chen, Wenhui Yang, Xiao Wang

**Affiliations:** Department of Orthopedics, Huaihe Hospital, Henan University, Henan, China.

**Keywords:** balance training, hip fracture, meta-analysis

## Abstract

**Background:**

We conducted this meta-analysis to analyze the effectiveness of balance training in improving postoperative rehabilitation outcomes in hip fracture surgery patients.

**Methods:**

The Cochrane Library, Web of Science, Embase, and PubMed electronic databases were searched from their inception to December 2018. We selected prospective clinical control analyses and high-quality randomized controlled trials (RCTs) following the inclusion standards. We used Stata 12.0 to perform the meta-analysis. Where possible, the standard mean difference (SMD) with the 95% confidence interval (CI) was determined using a random effects model.

**Results:**

Ten RCTs involving 955 hips (balance training = 487, control = 468) published between 2002 and 2019 were assessed for eligibility of inclusion in the meta-analysis. Balance training was shown to remarkably improve the aspects of quality of life associated with physical health (standard mean difference [SMD], 2.20; 95% CI, 1.63–2.78, *P* = .000), a fast gait speed (SMD, 1.01; 95% CI, 0.25–1.77, *P* = .009), and balance (SMD = 0.26, 95% CI: [0.12, 0.41], *P* = .000). Moreover, the balance training group showed increases in independence in activities of daily living (ADLs), performance task scores, and health-related quality of life (HRQoL) scores compared with the control group (*P* < .05).

**Conclusion:**

According to the present meta-analysis, balance training improves one's independence in activities of daily living, performance tasks, lower limb strength, gait, and total physical function compared with no balance training. More high-quality RCTs with large sample sizes are required for the identification of the best balance training program after hip fracture.

## 1 Introduction

Hip fractures are the most common injury in elderly patients.^[1]^ It was estimated that there were 16,518 hip fractures among adult Australians in 2006 to 2007, and this incidence is expected to rise due to the aging population.^[2,3]^ Nevertheless, individuals with hip fractures have more obvious postural sway, probably attributed to proprioception and muscular strength impairments.^[4]^ Moreover, independent walking is an important factor that affects quality of life after hip fracture. Thus, strategies that improve one's ability to walk independently are important for hip fractures.^[5]^

Studies have suggested that 2 years after a hip fracture, >39% of adult women die in long-term care facilities.^[6]^ In recent years, surgical interventions and other interventions following hip fracture have been shown to enhance the recovery process and reduce patient mortality and disability. In addition, balance deficits are the primary risk factors for falls.^[7]^ It is necessary to determine the best strategy for improving the functional outcomes of hip fracture patients.^[8]^

It has been shown that rehabilitation after hip fracture plays an important role in ensuring recovery after hip fracture and improving quality of life.^[9]^ Balance training can prevent elderly people from falling.^[10]^ Nevertheless, the influences of balance training on clinical results in hip fracture cases are unclear. Accordingly, meta-analyses and comparisons on balance training for hip fracture cases need to be conducted.

Currently, whether balance training is superior to a placebo or control intervention remains controversial due to the small number of published articles examining the efficacy of balance training for hip fracture patients. Therefore, we performed a meta-analysis of randomized controlled trials (RCTs) to evaluate the efficacy of balance training for hip fracture patients.

## 2 Materials and methods

This meta-analysis, which is presented according to the Preferred Reporting Items for Systematic Reviews and Meta-Analyses (PRISMA) statement guidelines,^[11]^ was conducted in accordance with the suggestions of the Cochrane Handbook for Systematic Reviews of Interventions. Ethical approval and patient consent are not required in a meta-analysis.

### 2.1 Search strategy

Two reviewers independently searched the following electronic databases for potentially relevant published studies: PubMed (1966 to December 2019), Embase (1974 to December 2019), and Web of Science (1990 to December 2019). We also used the Google search engine (December 2019) to search for additional eligible studies. The key words used included combinations of different terms and synonyms, as follows: ((((((((Fractures, Subtrochanteric) OR Subtrochanteric Fractures) OR Fractures, Intertrochanteric) OR Intertrochanteric Fractures) OR Fractures, Trochanteric) OR Trochanteric Fractures) OR Fractures, Hip)) OR (Femoral Neck Fracture OR Femur Neck Fractures OR Femur Neck Fracture OR “Femoral Neck Fractures”[Mesh]) AND (((((Training, Circuit) OR Circuit Training) OR Exercises, Circuit-Based) OR Exercise, Circuit-Based) OR balance training). We initially assessed the titles and abstracts of the articles identified in the search and then performed a careful review of the full texts. To maximize the scope and relevance of our search, we also used the backward chaining method to search the references of the retrieved papers.

### 2.2 Inclusion criteria

Studies were considered eligible for the meta-analysis if they met the following criteria: the population included patients with hip fractures, the intervention was balance training before or after the operation, the comparison included a placebo or control group, the outcomes included the total role, gait speed, lower limb strength, independence in activities of daily living (ADLs), performance task scores and health-related quality of life (HRQoL), and the study design was an RCT. The exclusion criteria for this study were as follows: animal studies; non-RCTs; and case reports, commentary papers, and correspondence articles. Disputes between the 2 reviewers will be settled through discussion or consultation with a third reviewer.

### 2.3 Data extraction

Two review authors will extract the data with a standard data extraction form independently. The collected data include the duration of follow-up, surgical procedure, number of hips affected, sample size and demographics of the participants, inclusion and exclusion criteria, study design, authors, publication date, and the participants’ physical health, fast gait speed, balance, ADLs, performance task scores, and HRQoL scores. The data were recorded by 2 reviewers separately, and disagreements were analyzed until a consensus was made. Kappa values were used to measure the degree of agreement between the 2 reviewers and were rated as follows: 0.40 to 0.59 indicated fair agreement, 0.60 to 0.74 indicated good agreement, and ≥0.75 indicated excellent agreement.

### 2.4 Risk posed by bias and quality assessment

The quality of the articles was assessed by the risk of bias table in Review Manager 5.0 (Cochrane Collaboration, 2011).^[12]^ Seven criteria were used in the evaluation: random sequence generation, allocation concealment, blinding of the participants and personnel, blinding of the outcome assessment, incomplete outcome data, selective reporting, and other bias. In addition, funnel plots were generated and Begg test and Egger test were conducted to estimate the degree of potential publication bias.

### 2.5 Statistical analysis

Meta-analysis was performed using Stata 12.0 software (Stata Corp., College Station, TX). Due to the diversity in the clinical or methodological characteristics, we pooled the results using a random effects model, with the standard mean differences (SMD) for the continuous outcome data, and calculated the 95% confidence intervals (CIs) and 2-sided *P* values for each overall effect size. Statistical heterogeneity among all the included articles was evaluated using the chi-square test and *I*^2^ statistic. A *P* value of <.05 was considered to indicate statistical significance. Publication bias was evaluated using a funnel plot, and sensitivity analyses were performed with Stata 12.0 (Stata Corp., College Station, TX). Funnel plots and Begg test were performed to identify publication bias. Subgroup analysis was performed according to the patients’ age, risk of bias, frequency training, and follow-up duration.

## 3 Results

### 3.1 Search results

**Figure 1:**
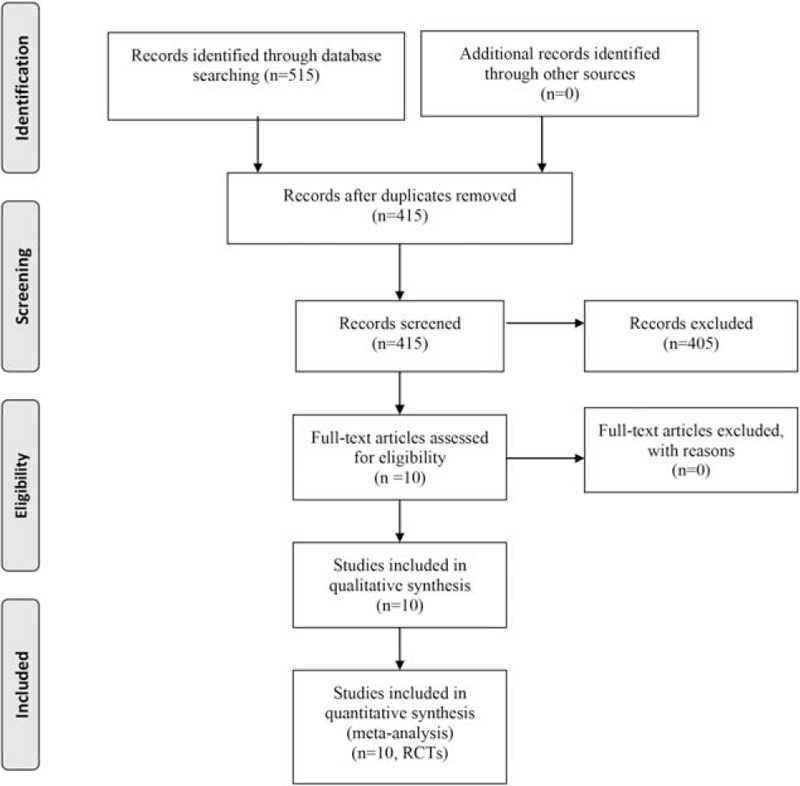
Flow diagram of the study selection process.

The initial search yielded 515 studies; 415 were selected for the eligibility assessment after the exclusion of duplicate publications. After detailed evaluations, which included reading the abstract, checking the study design, and examining the data in the papers, 405 papers were excluded because of the study design. Finally, 10 studies^[13–22]^ with 955 hips (balance training = 487, control = 468) met the eligibility criteria and were included in the current meta-analysis. The literature search and screening process is shown in Fig. 1.

### 3.2 General characteristics of the included studies

All the articles were published between 2002 and 2019. The sample size ranged between 13 and 120. There were 487 patients in the balance training group and 468 in the control group in the studies. Table 1 lists the names of the first authors, years of publication, study designs, sample sizes, patients’ sexes, patients’ mean ages, intervention start dates, intervention durations, outcomes, and follow-up times.

**Table 1:**
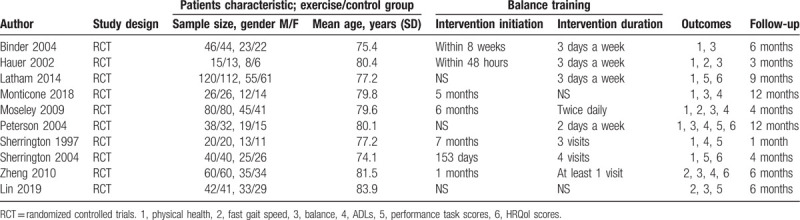
General characteristic of the included RCTs.

### 3.3 Risk of bias

**Figure 2:**
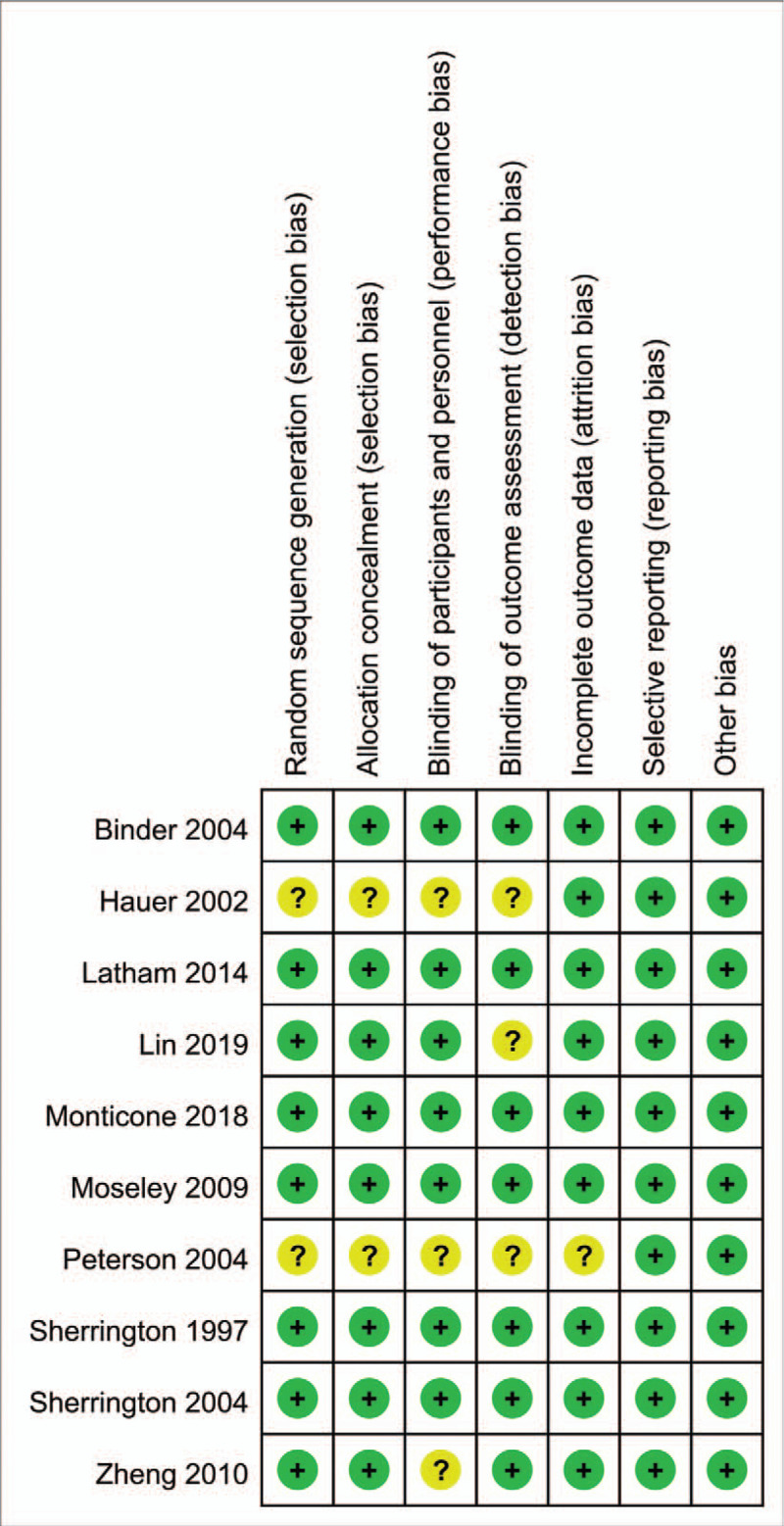
Methodological quality assessment of each included study. Legend for the types of marks: “+”, low risk of bias; “−”, high risk of bias; “?”, unclear risk of bias.

**Figure 3:**
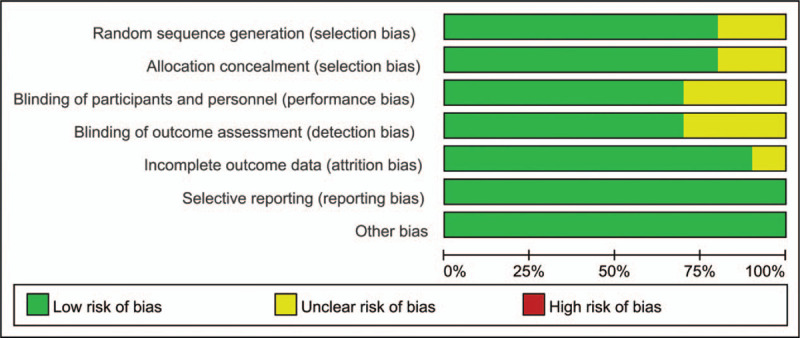
Risk of bias graph of the included studies.

Figures 2 and 3 show a summary and graph of the risk of bias, respectively. Three studies were considered to have a low risk of bias, 3 studies were considered to have a high risk of bias, and the remaining studies were considered to have an unambiguous risk of bias. The kappa value between the reviewers was 0.852, indicating an excellent degree of agreement between the 2 reviewers.

### 3.4 Results of the meta-analysis

#### 3.4.1 Physical health

**Figure 4:**
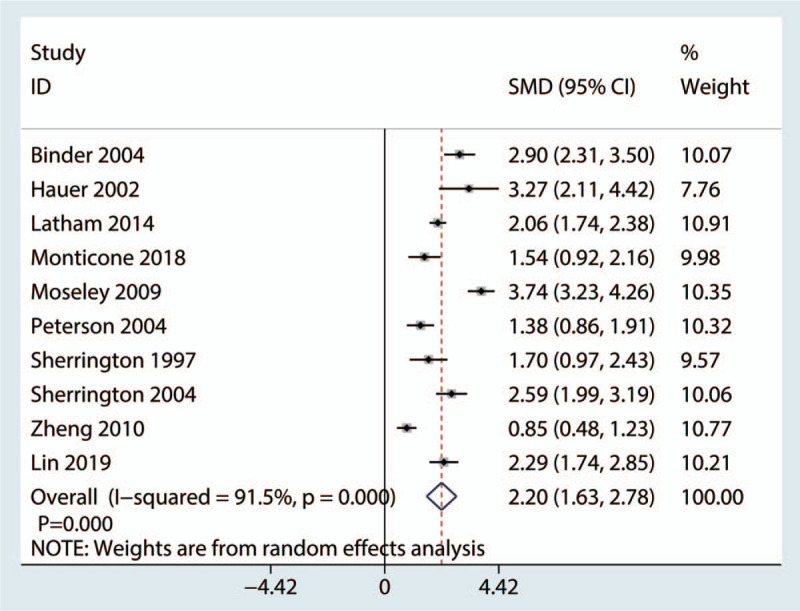
Forest figure used to compare physical health between the balance training and control groups.

A total of 10 studies (955 patients) provided data on the patients’ physical health. The pooled data showed that balance training was associated with improved physical health compared with the control treatment (SMD, 2.20; 95% CI, 1.63–2.78, *P* = .000). Significant heterogeneity was detected in these studies (*P* = .000; *I*^2^ = 91.5%). Thus, a random effects model was used (Fig. 4).

#### 3.4.2 Fast gait speed

**Figure 5:**
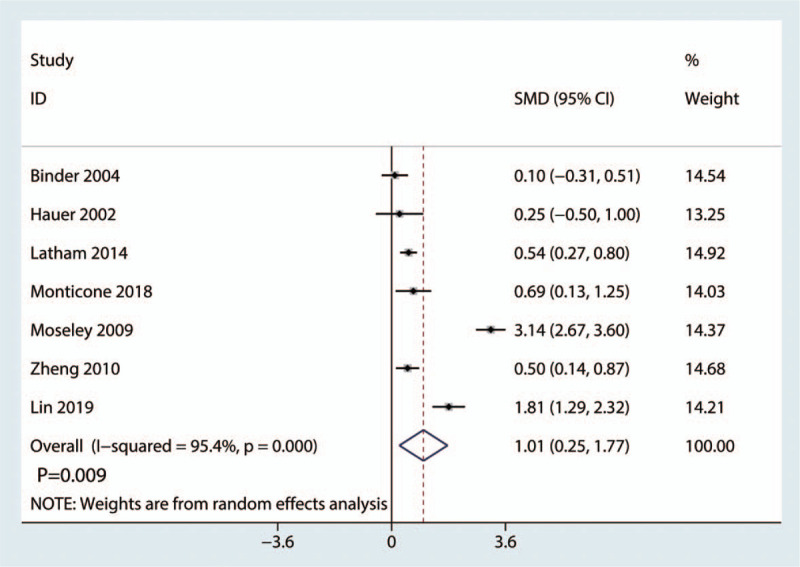
The forest plot of the fast gait speed in the balance training group versus the control group.

A total of 7 studies (765 patients) provided data on the fast gait speed. The pooled data showed that balance training was associated with an increase in the fast gait speed compared with the control treatment (SMD, 1.01; 95% CI, 0.25–1.77, *P* = .009). Significant heterogeneity was detected in these studies (*P* = .000; *I*^2^ = 95.4%). Thus, a random effects model was used (Fig. 5).

#### 3.4.3 Balance

**Figure 6:**
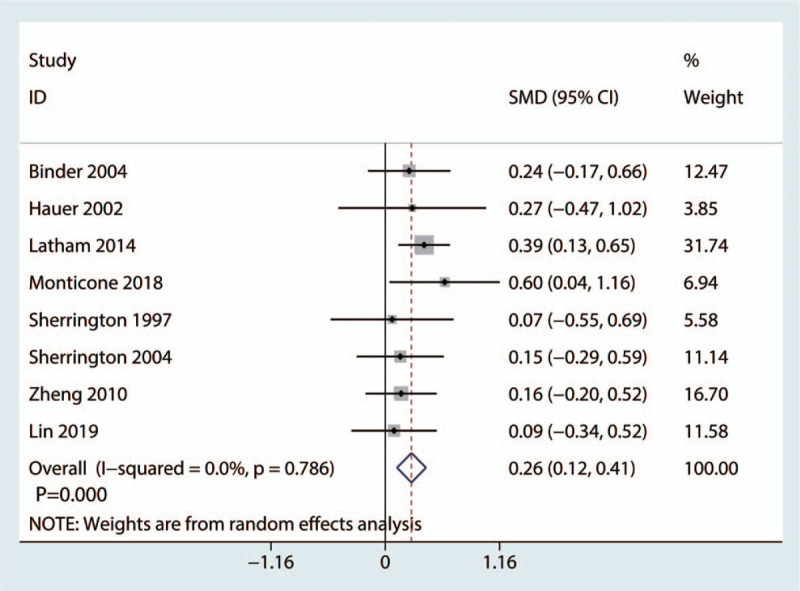
Forest plot of the balance performance of the control and balance training groups.

Eight studies covering 762 cases reported results regarding balance. No heterogeneity was found among the 8 studies (*I*^2^ = 0.0%; *P* = .786, Fig. 6). Thus, a random effects model was used. In addition, the meta-analysis suggested that balance training improved balance performance compared with the control treatment (SMD = 0.26, 95% CI: [0.12, 0.41], *P* = .000; Fig. 6).

#### 3.4.4 ADLs

**Figure 7:**
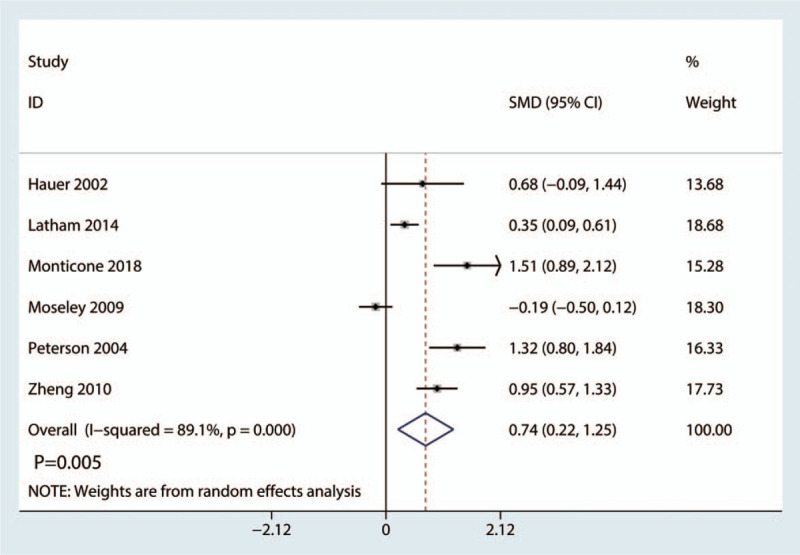
Forest plot comparing the individuals’ level of independence in performing ADLs between the reference and balance training groups. ADLs = activities of daily living.

Six studies covering 662 cases reported the patients’ level of independence in performing ADLs after hip surgery. The 6 studies (*I*^2^ = 89.1%; *P* = .000, Fig. 7) demonstrated high heterogeneity. A random-influences model was used. In addition, the meta-analysis suggested that balance training improved patients’ level of independence in performing ADLs compared with the control treatment (SMD = 0.74, 95% CI: [0.22, 1.25], *P* = .005; Fig. 7).

#### 3.4.5 Performance task scores

**Figure 8:**
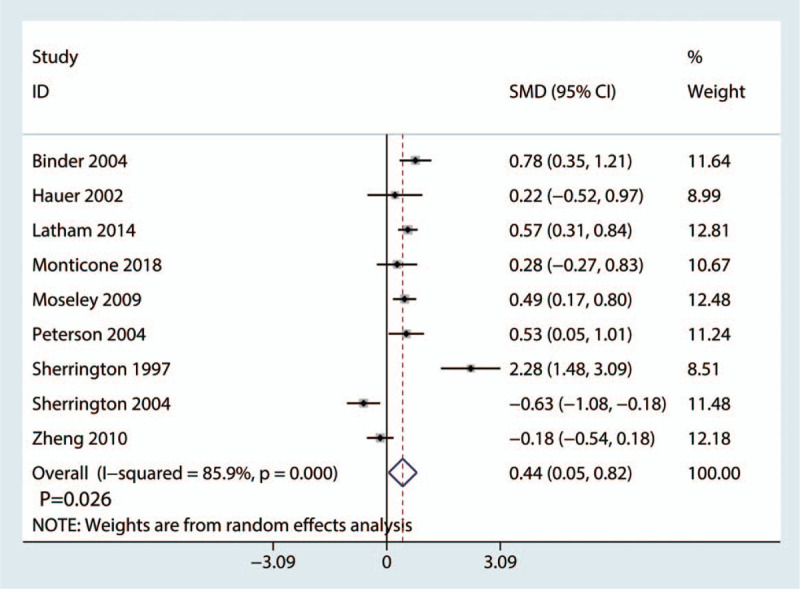
Forest plot comparing the performance task scores between the reference and balance training groups.

Nine studies covering 872 cases reported performance task scores after hip surgery. The 9 studies demonstrated mild heterogeneity (*I*^2^ = 48.1%, *P* = .051, Fig. 8). Thus, a random-influences model was used. The meta-analysis suggested that balance training can noticeably improve the performance task scores (SMD = 0.44, 95% CI: [0.05, 0.82], *P* = .026; Fig. 8).

#### 3.4.6 HRQoL scores

**Figure 9:**
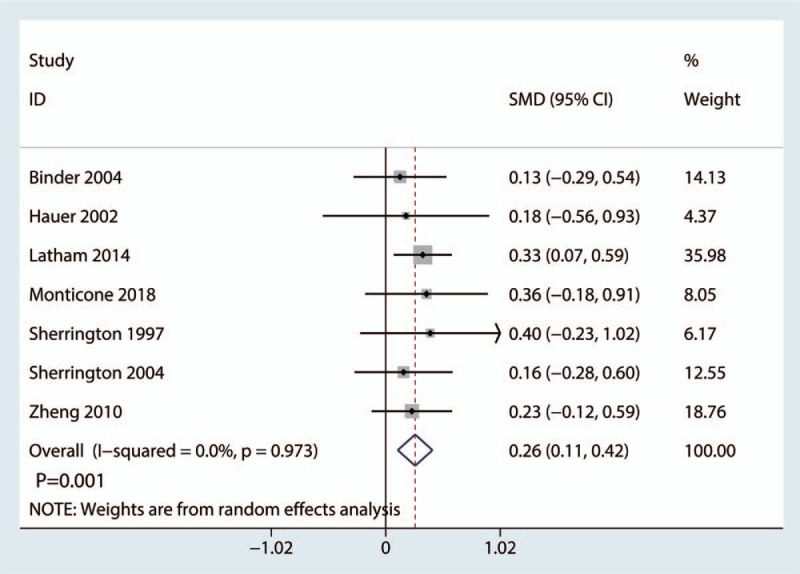
Forest plot comparing the HRQoL scores between the reference and balance training groups. HRQoL = health-related quality of life.

Seven studies covering 642 cases reported HRQoL scores after hip surgery. No heterogeneity was found among the 7 studies (*I*^2^ = 0.0%, *P* = .973; Fig. 9). Accordingly, we used a fixed effects model. The meta-analysis suggested a remarkable difference between the balance training and control groups (SMD = 0.26, 95% CI: [0.11, 0.42], *P* = .001; Fig. 9).

#### 3.4.7 Publication bias, sensitivity analysis, and subgroup analysis

**Figure 10:**
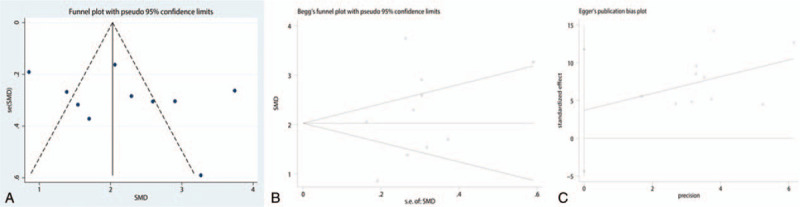
A, Funnel plot of the total role of the reference and balance training groups; B, Begg test results for total role of the reference and balance training groups; C, Egger test results for total role of the reference and balance training groups.

**Figure 11:**
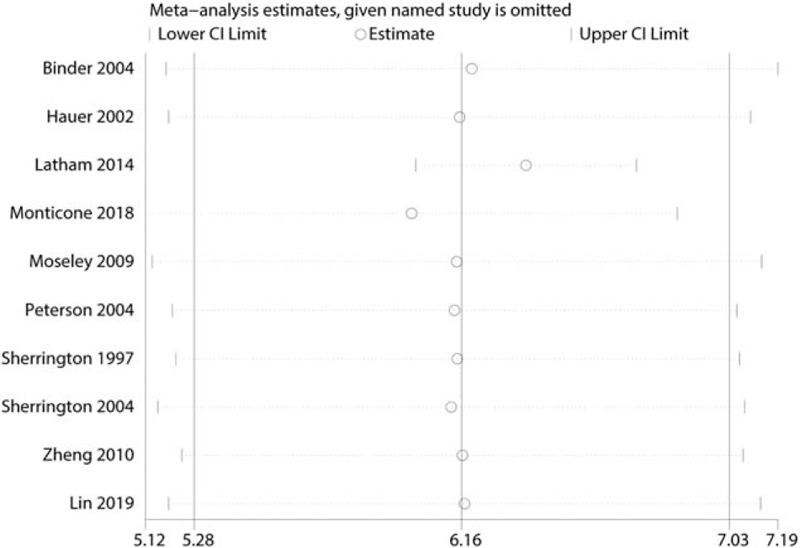
Sensitivity analysis for the total role of the reference and balance training groups.

To conduct a meta-analysis on the effects of balance training on physical health, the publication bias of the studies was assessed; the shape of the funnel plot showed no evidence of asymmetry (Fig. 10A), and the formal statistical tests did not indicate publication bias (Egger test, *P* = .589, Fig. 10B; Begg test, *P* = .921, Fig. 10C). We also conducted a sensitivity analysis to further explore the sources of heterogeneity. The results indicated that after each study was removed in turn, the overall effect size remained unchanged (Fig. 11). We performed subgroup analysis based on patient age, risk of bias, frequency training, and follow-up duration (Table 2). The subgroup analysis of physical health indicated that balance training for >3 days a week was more effective than that for ≤2 days a week in improving the physical health of hip fracture patients (1.72 vs 3.36).

**Table 2:**
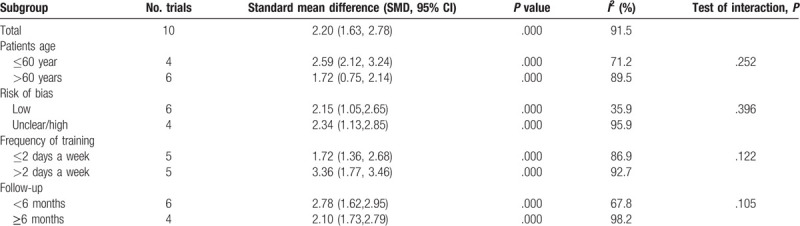
Subgroup analysis for overall function between balance training and control group.

## 4 Discussion

### 4.1 Main findings

The results here suggested that balance training improves individuals’ physical health, fast gait speed, balance performance, independence in performing ADLs, performance task scores, and HRQoL scores. According to the subgroup results, training at a high frequency outperforms training at a low frequency for enhancing one's physical health.

### 4.2 Comparison with other meta-analyses

Only one related meta-analysis has been conducted.^[23]^ The discrepancies between this study and the existing ones are noteworthy. A range of training frequencies should be studied. This meta-analysis carried out a subgroup analysis and assessed the risk of publication bias for all the studies. Doma et al^[24]^ reported that balance training enhanced functional outcome measures, balance-specific performance, and walking capacity for elderly people who underwent overall knee arthroplasty.

### 4.3 Implications for clinical practice

The present meta-analysis suggested that balance training can noticeably improve individuals’ physical health, fast gait speed, balance performance, independence in performing ADLs, performance task scores, and HRQoL scores after hip fracture. In addition, training at a high frequency was suggested. Latham et al^[15]^ suggested that home-based functionally oriented exercise programs lead to modest enhancement in physical function at 6 months. Nevertheless, additional studies need to be conducted to determine the clinical significance of balance training. Chen et al^[25]^ found that a home-based exercise program had a positive, although not significant, effect on physical function after hip fracture.

Existing studies have suggested that patients show less favorable functional results after hip fractures.^[26]^ After experiencing a fracture, individuals are at high risk of entering a vicious cycle in which individuals experience a fear of falling, post-fracture pain, and myasthenia.^[27]^ Existing exercise studies that have used rigorous specialized supervision and equipment have demonstrated a remarkable capacity for adults with hip fracture to improve after balance training.^[13,28]^ We assessed 9 RCTs and found that balance training positively enhances the total role, gait speed, and lower limb strength. Individuals’ levels of independence in performing ADLs were compared between the balance training and reference groups. The balance training group outperformed the reference group in terms of the level of independence in performing ADLs. In addition, the balance training had higher HRQoL scores than the reference group. Combs et al^[29]^ suggested that balance training can noticeably improve the aspect of one's quality of life associated with his or her health.

In general, the present research had a number of strengths, which are as follows: a comprehensive retrieval strategy was employed to lower the risk of publication bias, and sensitivity and subgroup analyses were conducted to better interpret the present results.

Some weaknesses of the present study cannot be ignored. First, the number of included trials was limited, which may lead to limited generalizability and more uncertainty. Due to the lack of primary studies with a relatively long follow-up period, it is difficult to determine the statuses of the hip fractures treated by balance training after 1 year. Hence, other trials with longer follow-up periods should be performed in the future. The optimal strategy for balance training was not explored in the included studies.

### 4.4 Conclusion

Balance training improved individuals’ independence in performing activities of daily living, performance task scores, lower limb strength, gait, and total physical function compared with the control treatment. A large number of high-quality RCTs are required to identify the best type of balance training for individuals after hip fracture.

## Author contributions

**Conceptualization:** Xinxin Chen.

**Resources:** Xiao Wang.

**Software:** Xiao Wang.

**Visualization:** Wenhui Yang.

**Writing – original draft:** Wenhui Yang.

**Writing – review & editing:** Xinxin Chen.
